# Revising REACH guidance on information requirements and chemical safety assessment for engineered nanomaterials for aquatic ecotoxicity endpoints: recommendations from the EnvNano project

**DOI:** 10.1186/s12302-017-0111-3

**Published:** 2017-03-09

**Authors:** Steffen Foss Hansen, Sara Nørgaard Sørensen, Lars Michael Skjolding, Nanna B. Hartmann, Anders Baun

**Affiliations:** Department of Environmental Engineering, Technical University of Denmark, DTU-building 115, 2800 Kgs. Lyngby, Denmark

**Keywords:** ECHA guidance, EnvNano, Nanomaterials, Ecotoxicity testing

## Abstract

The European Chemical Agency (ECHA) is in the process of revising its guidance documents on how to address the challenges of ecotoxicological testing of nanomaterials. In these revisions, outset is taken in the hypothesis that ecotoxicological test methods, developed for soluble chemicals, can be made applicable to nanomaterials. European Research Council project EnvNano—Environmental Effects and Risk Evaluation of Engineered, which ran from 2011 to 2016, took another outset by assuming that: “*The behaviour of nanoparticles in suspension is fundamentally different from that of chemicals in solution”*. The aim of this paper is to present the findings of the EnvNano project and through these provide the scientific background for specific recommendations on how ECHA guidance could be further improved. Key EnvNano findings such as the need to characterize dispersion and dissolution rates in stock and test media have partially been addressed in the updated guidance. However, it has to be made clear that multiple characterization methods have to be applied to describe state of dispersion and dissolution over time and for various test concentration. More detailed information is called for on the specific characterization methods and techniques available and their pros and cons. Based on findings in EnvNano, we recommend that existing algal tests are *supplemented* with tests where suspensions of nanomaterials are aged for 1–3 days for nanomaterials that dissolve in testing media. Likewise, for daphnia tests we suggest to supplement with tests where (a) exposure is shortened to a 3 h pulse exposure in daphnia toxicity tests with environmentally hazardous metal and metal oxide nanomaterials prone to dissolution; and (b) food abundance is three to five times higher than normal, respectively. We further suggest that the importance of considering the impact of shading in algal tests is made more detailed in the guidance and that it is specified that determination of uptake, depuration and trophic transfer of nanomaterials for each commercialized functionalization of the nanomaterials is required. Finally, as an outcome of the project a method for assessing the regulatory adequacy of ecotoxicological studies of nanomaterials is proposed.

## Background

The European Chemical Agency (ECHA) is in the process of revising nano-related appendices in the existing Technical Guidance to accompany the ongoing revisions to the REACH annexes VIII, IX and X on information requirements [24]. The first editions of these appendices were published in 2012 [19–21] and included the recommendations made in the REACH implementation projects on nanomaterials and information requirements and on chemical safety assessment (also known as RIP-oN 2 and RIP-oN 3, respectively) [5, 37]. While other groups of chemicals (petroleum substances, metal and inorganic substances) are mentioned within the main Technical Guidance Documents [22], a different approach was taken for nanomaterials, resulting in so far six nanospecific appendices. The literature on environmental fate and effects of nanomaterials has expanded vastly, since the latest version of the nano-related appendices to REACH Technical Guidance in 2012 (as summarized/reviewed by Peijnenburg et al. [50], Juganson et al. [59]) and the scientific progress must be incorporated into the upcoming revisions. For nanoparticles, ecotoxicological testing presents specific challenges related to the altered behavior and properties of nanoparticle suspensions compared to chemical solutions [74] and possible strategies for coping with these challenges and adapting testing guidelines have been investigated within numerous European research projects, e.g., MARINA, NanoValid and NanoREG. From these projects, a number of recommendations have been made and overview articles have been published [e.g., 9, 44, 45]. In most of these projects, an incremental approach to revision of the OECD guidelines has been applied, assuming that the test methods, developed for soluble chemicals, can be made applicable to nanomaterials through methodological adaptation. The properties of nanomaterials, however, clash with the fundamental prerequisite of many of these test methods, i.e., that the test substance is water soluble, implying that it distributes in the test system by molecular diffusion. Since this has been repeatedly proven not to be the case for nanomaterials (for overviews see, e.g., [64]), the European Research Council project EnvNano—Environmental Effects and Risk Evaluation of Engineered, which ran from 2011 to 2016, took another outset by assuming that: “*The behaviour of nanoparticles in suspension is fundamentally different from that of chemicals in solution. Therefore, all modifications of existing techniques that do not take this fact into account are bound to have a limited sphere of application or in the worst case be invalid, give meaningless and/or misleading results.”* [34].

The aim of this paper is to present the findings of the EnvNano project and through these provide the scientific background for specific recommendations for testing the aquatic toxicity of nanoparticles to serve as inputs to the revisions of ECHA’s appendices to the Technical Guidance Documents for chemical safety assessment.

In the following, we first provide a short summary of the current status on nanomaterials in ECHA’s guidance documents, list the key findings from the EnvNano project and analyze where and how these findings could be integrated into the REACH Technical Guidance appendices and REACH Annexes. We also indicate how and where these recommendations link to the text in the main Technical Guidance documents and, where relevant, highlight the need for additional appendices to address topics not covered by the current documents. The recommendations are formulated such that they can be directly integrated into the existing guidance text.

## Background: current status on nanomaterials in REACH and ECHA’s guidance on testing of nanomaterials

ECHA provides a series of guidance documents to assist manufacturers and importers that have to meet different obligations under REACH on, e.g., registration, substance identification, chemicals safety assessment in order to**”**…facilitate the implementation of REACH by describing good practice on how to fulfil the obligations…” [25] that REACH places on industry. These documents have been developed with the participation of many different stakeholders, e.g., industry, Member States and NGOs [25].

This includes guidance on Information Requirements (IR) and Chemical Safety Assessment (CSA) which describes how the information requirements under REACH can be met by industry when it comes to documenting substance properties, exposure, use, and risk management in their chemical safety assessments. The guidance covers the collection and assessment of available information regarding the physical and chemical properties of a given substance; the identification of data gaps and the generation of the additional information required to fill the data gaps [26].

The guidance in itself consists of two major parts: Concise guidance (Part A to F) and supporting reference guidance (Chapters R.2 to R.20). These reference guidance chapters cover information requirements (R.2–R7), dose [concentration]—response characterization (R.8–R.10), PBT/vBvP assessment (R.11), exposure assessment (R.12–R.18), uncertainty analysis (R.19) and R.20 provides an explanation of terms [26]. R.7 and R.10 are particularly relevant for to nanomaterials and ecotoxicity. R.7 on endpoint-specific guidance consists of 3 parts, namely R.7a on physico-chemical properties and human health endpoints, R.7b on aquatic ecotoxicity and (bio)degradability and R.7c on aquatic and terrestrial bioaccumulation, avian toxicity, effects of terrestrial organisms, toxico-kinetic, and substances requiring special considerations regarding testing and exposure [22, 23, 27]. R.10 describes how to calculate Predicted No Effect Concentrations (PNECs) for water, sediment, etc. and describes the process of how to derive PNECs including data to be used and the how to evaluate and interpret data [18].

The six appendices to the reference guidance were prepared in 2012 to address the lack of nanospecific guidance. These documents include recommendations on physico-chemical characterization (appendix to R.7a), aquatic ecotoxicity and (bio)degradability (appendix to R.7b), aquatic bioaccumulation and terrestrial ecotoxicity (appendix to R.7c), dose–response characterization, human toxicity (appendix to R.8), dose–response characterization, environment (appendix to R.10) and occupational exposure (appendix to R.14), respectively. Some of the guidance in current appendices is quite extensive, such as the guidance on how to determine particle size distribution, specific surface area, and particle shape in the appendix to R.7a. On the other hand, very limited guidance is given on water solubility in that same appendix, on ecotoxicological testing in the appendix to R.7b or on dose–response characterization (environment) (appendix to R.10).

In 2015, ECHA prepared a series of draft documents updating some of the nanospecific appendices and these are currently subject to an ongoing guidance consultation [24]. For Appendix R7-1 on Chapter R7a, the suggested updates include important aspects to consider in sample preparation such as taking the test media characteristics into account in the preparation and dispersion of the tested nanomaterials and using another dose metrics than mass based when available. It furthermore entails the recommendations to measure the rate and extent of dissolution; not to waiver information requirements based on high insolubility only and not to use n-octanol/water partition coefficient for nanomaterials without justification [28].

With respect to the recommendations for ecotoxicological endpoints for nanomaterials, substantial revisions have been made in the draft documents currently out for consultation [29, 30]. This is especially the case for aquatic pelagic toxicity. General issues to be considered during test planning include defining representative controls, dissolution rate and potential ion release, agglomeration behavior, degradation and transformation, selection of the exposure regimes, frequency of concentration measurements, use of mass-based metrics, and nanospecific measurements. General issues are also raised with regard to preparation of stock solutions, e.g., reporting and justifying direct preparation of stock solutions versus use of dispersion protocols, and dispersion stability and test media, e.g., considering agglomeration behavior and particle stability in the test medium. In the nanospecific guidance [28], text has been added to the 2012 version [19] stating that synthetic dispersants should not be used to prepare stock solutions; that media characteristics such as pH and ionic strength should be provided, and that exposure concentration should be measured. Furthermore, it is stated that dissolution rate should be considered instead of equilibrium solubility for nanomaterials. If the nanomaterial dissolves quickly and has a high solubility, no further specific nanomaterial considerations are needed. In the opposite case, for non- or slowly dissolving nanomaterials, long-term toxicity testing is advised. A range of additional parameters specific for aquatic toxicity are recommended for reporting: For fish testing, these include reporting fish brain pathology, animal behavior and histopathology of fish whereas they for daphnids testing include reporting of mechanical effects, e.g., adherence to the organisms, blocking of respiratory organs and, finally, for algal test quantification of effects on color or shading and fluorescence measurement of chlorophyll extracts. With regard to aquatic bioaccumulation, it is repeated from the earlier version that QSARs have a very limited use when assessing aquatic bioaccumulation. Generally, OECD TG 305 Bioaccumulation in Fish is applicable for nanomaterials when using the dietary route of exposure. However, the aqueous route of uptake resulting in BCF is, for most nanomaterials, not applicable due to non-equilibrium between the organism and the water phase and difficulties in keeping aqueous exposure constant. Currently, a new OECD Guidance is being developed that will provide further clarification on how to measure and quantify the accumulation potential of nanomaterials in fish [21, 30]. ECHA currently recommends that the use of bioaccumulation in sediment through the OECD TG 315 and OECD TG 317 can be used as a weight of evidence in bioaccumulation assessment. It is highlighted that for any test to be performed with nanomaterials test concentrations has to be monitored throughout the test duration to account for changes in dispersion and agglomeration/aggregation characteristics [30].

## Main findings of the EnvNano project

When analyzing the ECHA guidance on testing of nanomaterials and mirroring these up against the findings in EnvNano, it is clear that some of the findings of EnvNano have already been integrated in the suggested revisions of the nanospecific appendices [41, 60, 75; Sørensen et al. 2015; 43] whereas others also contain information of relevance for the revisions [e.g., 14, 54, 70, 72–74]. There are several sections in the guidance that could and should be updated and/or made more detailed ranging from how specific OECD technical guidelines should be used to how to evaluate the reliability of the experimental data for use in chemical safety assessments. Table 1 provides an overview of the possibilities for further improvements that we have identified in the current appendices and suggested changes with reference to the following sections, where we provide more detailed description of these as well as ten recommendations.

**Table 1:** Overview of the possibilities for further improvements of the ECHA guidance identified in the current appendices and the changes that we suggest

ECHA TG	Section in ECHA TG	Page	Room for improvement in the current text	Section in this paper addressing this issue	EnvNano recommendation(s) related to this issue
Nanospecific Appendix R.7a v2	1.1 General advice on how to perform nanomaterials ecotoxicity and fate testing	6	No mentioning of the importance of understanding and considering the pros and cons of various characterization methods in the listed prerequisites	– Appropriate nanomaterial dispersion is key for reliable ecotoxicity testing – Quantifying nanomaterial dissolution is crucial for disclosing ecotoxic effects – Nanomaterial surface reactivity is important as toxicity indicator	EnvNano recommendation # 1 on dispersion EnvNano recommendation # 2 on dissolution EnvNano recommendations # 3 on ROS
Nanospecific Appendix R.7a v2	1.1 General advice on how to perform nanomaterial ecotoxicity and fate testing	7	No mentioning of the importance of preparation of the stock solution to minimize agglomeration/aggregation	– Nanomaterial dispersion is key for reliable ecotoxicity testing	EnvNano recommendation # 1 on dispersion
Nanospecific Appendix R.7b v2	1.2.1 Aquatic pelagic toxicity	8	Could be more specific with regard to how to determine whether an NP dissolves fast or not	– Quantifying nanomaterial dissolution is crucial for disclosing ecotoxic effects	EnvNano recommendation # 2 on dissolution
Nanospecific Appendix R.7b v2	1.2.1.1 Test guidelines specificities for aquatic toxicity	9	Not sufficiently specific regarding OECD TG 201 on algal growth inhibition testing	– A shortened exposure may reduce nanomaterial transformations in ecotoxicity tests and elucidate nanomaterial-specific effects and exposure dynamics – Using freshly prepared nanomaterial-suspensions may underestimate toxicity – It is important to distinguish between physical and chemical effects in aquatic toxicity tests	EnvNano recommendation # 4 for acute algal tests with nanomaterials EnvNano recommendation # 5 on not only using a freshly nanomaterial-suspensions EnvNano recommendation # 6 on shading and physical effects
Nanospecific Appendix R.7b v2	1.2.1.1 Test guidelines specificities for aquatic toxicity	9	Not sufficiently specific regarding OECD TG 202 on *daphnia magna* acute toxicity testing	– Uptake and depuration depend on nanomaterial functionalization – It is important to distinguish between physical and chemical effects in aquatic toxicity tests	EnvNano recommendation # 8 for acute daphnia tests with nanomaterials EnvNano recommendations # 6 on shading and physical effects
Nanospecific Appendix R.7b v2	1.2.1.1 Test guidelines specificities for aquatic toxicity	9	Could be more specific regarding OECD TG 211 on *daphnia magna* chronic toxicity testing	– Toxicity and uptake is feeding dependent	EnvNano recommendation # 7 for long-term daphnia tests with nanomaterials
Nanospecific Appendix R.7c v2	2.1.1. Aquatic bioaccumulation	7	No consideration of integration of the nanomaterials into food sources and potential trophic transfer	– Trophic transfer is an important uptake pathway for nanomaterials	EnvNano recommendation # 9 for bioaccumulation tests with nanomaterials
R.7b Endpoint specific guidance	7.8.2 Information requirements for aquatic pelagic toxicity	15	Limited nanospecific relevance of existing general information requirements	– Appropriate nanomaterial dispersion is key for reliable ecotoxicity testing – Quantifying nanomaterial dissolution is crucial for disclosing ecotoxic effects – Nanomaterial surface reactivity is important as toxicity indicator	EnvNano recommendation # 1 on dispersion EnvNano recommendation # 2 on dissolution EnvNano recommendation # 3 on ROS
	7.8.4.1 Evaluation of available information on aquatic pelagic toxicity	22–23	Klimisch scoring does not take nanospecific properties into account	– Data selection for dose–response assessment derivation should be structured, reproducible and transparent and support use on non-guideline data	EnvNano recommendation # 10 on evaluation of data

### Appropriate nanomaterial dispersion is key for reliable ecotoxicity testing

**Fig. 1:**
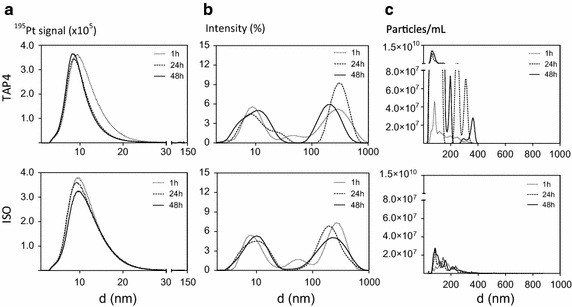
Influence of incubation periods and media on size distributions determined by different methods. Size distributions after different incubation periods (1–48 h) for Pt nanoparticles suspended in TAP4 (4× diluted tris–acetate-phosphate) medium (*top row*) and ISO medium (*bottom row*) determined by different methods. **a** Suspensions of 4 mg Pt/L analyzed by Asymmetric Flow Field-Flow Fractionation (AsFlFFF); **b** Suspensions of 30 mg Pt/L analyzed by Dynamic Light Scattering (DLS); **c** Suspensions of 80 mg Pt/L analyzed by Nanoparticle Tracking Analysis (NTA). Reprinted with permission from Sørensen et al. [78]. Copyright (2016) American Chemical Society

Many of the standard test guidelines for aquatic ecotoxicity testing require that test substance is tested either as fully dissolved or as a stable suspension, and that tests are carried out after equilibrium with the surrounding test system has been reached. This fundamental prerequisite will be violated for most nanomaterials since they are dispersed rather than dissolved and stable suspensions are inherently difficult to obtain and maintain during incubation [41]. The reliability of test results obtained in tests of instable suspensions is low, with time and concentration-dependent processes like dissolution, agglomeration and sedimentation ongoing during the incubation [6, 65]. Several ecotoxicological studies have shown how agglomeration/aggregation is influenced by nanomaterial concentration and characteristics such as size, shape and coating, but also properties of the medium including pH, ionic strength, dissolved oxygen and NOM [14, 15, 31, 51, 53, 85]. An increase in particle concentration will theoretically increase the collision rate between particles and, thus, lead to more agglomeration/aggregation and less stable suspension as a function of the tested concentrations [6]. This has been demonstrated experimentally for, e.g., silver and iron nanomaterials [6, 62, 63]. This implies that, if the process occurring is (irreversible) aggregation, the nanoparticles would still be present in an aggregated form even upon dilution to the required test concentrations. To minimize aggregation in test suspensions, steps should hence be taken already in the preparation of the stock dispersion to minimize agglomeration/aggregation as well as in the following steps when diluted into test media, and should be monitored throughout the test using relevant techniques. In a study by Sørensen et al. [78], Pt nanoparticles were found to undergo rapid (within 1 h) and substantial agglomeration/aggregation in two algal media over 48 h. Using three different methods, namely Asymmetric Flow Field-Flow Fractionation (AsFlFFF) (4 mg Pt/L), Dynamic Light Scattering (DLS) (30 mg Pt/L) and Nanoparticle Tracking Analysis (NTA) (80 mg Pt/L), information on size distribution was obtained (see Fig. 1). AsFlFFF and DLS revealed a size peak of 10 nm (Fig. 1a, b), which was not identified by NTA having as the size detection limit is higher than 10 nm. According to NTA and DLS, the agglomerates were in the size range of 50–400 nm for both media, whereas such agglomerates were not identified by AsFlFFF. NTA, on the other hand, provided information on the number of agglomerates formed, which was not obtained by AsFlFFF and DLS, showing the number of PtNP agglomerates (>50 nm) increased almost three orders of magnitude in the TAP4 medium, compared to the ISO medium (Fig. 1c). This is in agreement with the studies on Ag nanoparticles [7, 47, 77]. Hence, it is clear that each of the three methods has strengths and limitations that have to be considered and taken into account when interpreting the data but only by using multiple characterization methods a clearer impression of the state of the dispersion can be obtained.

Aggregation of suspended nanoparticles is often termed homo-aggregation, whereas the aggregation of nanomaterials with other suspended particles including biota, organic and inorganic entities is referred to as hetero-aggregation [40]. There have been many studies on influencing factors in aquatic ecotoxicity tests (see review by Petersen et al. [61]. However, few studies have investigated the temporal changes in agglomeration/aggregation in standard ecotoxicity test media [68]. For Ag nanoparticle suspensions stabilized in various ways, substantial and rapid agglomeration/aggregation was reported immediately after suspension in OECD M7 test medium and increasing after 24–72 h, corresponding to the duration of an acute toxicity test with *D. magna* [68, 69, 80]. In M7 medium, increasing hydrodynamic diameters of nanoparticles have also been found during 24 h for TiO_2_ nanoparticles [14], Au nanoparticles [72] and Ag nanoparticles [79] with or without stabilizing agents. Although standard aquatic toxicity tests generally constitute relatively simple systems of synthetic freshwater (i.e., no sediment, natural particles), the biota and media alone can generate macromolecules/particles that may interact with nanomaterials. Agglomeration/aggregation may potentially result in sedimentation of particles due to gravitational settling of the formed agglomerates/aggregates. As agglomeration/aggregation and sedimentation are related, the factors influencing agglomeration will also impact sedimentation [65]. Sedimentation will be a bigger issue for non-agitated tests, such as the *D. magna* immobilization test. For CuO nanoparticles, the water phase exposure concentration in *D. magna* immobilization tests was observed to be reduced by more than 50% within few hours, due to sedimentation [79].

#### Recommendation # 1

Nanoparticle dispersion stability should be measured using multiple characterization methods. This entails characterization of the nanoparticle transformation processes relevant to the nanoparticles and test in question, such as agglomeration/aggregation, dissolution and sedimentation over the exposure duration of the ecotoxicity test. Various analytical methods are available for the characterization of these processes, including but not limited to: dynamic light scattering techniques (DLS and NTA), Field-Flow Fractionation (FFF), ultracentrifugation, membrane filtration techniques, UV–Vis and electron microscopy (EM). The limitations, pros and cons of the applied methods must be clearly described and the method should be selected to supplement each other to ensure reliable ecotoxicity testing. We furthermore recommend that concentrations of stock suspensions are chosen as close to the highest tested concentration as practically possible to minimize agglomeration/aggregation.

### Quantifying nanomaterial dissolution is crucial for disclosing ecotoxic effects

**Fig. 2:**
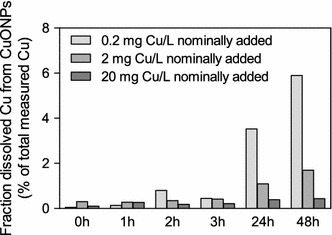
Time and concentration dependent dissolution of CuO nanoparticles suspended in modified M7. This figure shows the fraction of dissolved copper measured in the supernatant of ultracentrifuged CuO NP suspensions (of nominal concentrations 0.2, 2 and 20 mg Cu/L) determined 0, 1, 2, 3, 24 and 48 h after suspending the CuO nanoparticles (shown as percent of the total measured copper concentration at 0 h). All measured Cu concentrations are corrected for the Cu added as micro-nutrient to the medium. Reprinted with permission from Sørensen et al. [79]

Dissolution and dissolution kinetics has repetitively been observed to be crucial for understanding ecotoxic effects of nanoparticles [74]. In a study by Sørensen et al. [79], the behavior of CuO nanoparticles in OECD M7 medium was quantified at different concentrations over a timescale of 48 h, corresponding to an acute immobility test. Overall, the dissolution was found to increase with incubation time and decrease (percentwise) with CuO NP concentration (see Fig. 2). These findings correspond with results by other studies on CuO NP as well as studies on Ag nanoparticles [8, 6].

The presence of dissolved chemical species occurring concomitantly with nanoparticles results in a complex exposure scenario in which metal ions, metal complexes and particles are subject to different transformation processes, and possibly entail different bioavailability and toxicity mechanisms. These processes are also likely influenced by the presence of test organisms [11], e.g., through release of organic substances such as algal exudates. Ideally, the exposure and effects of these different fractions should be differentiated, but this is in practice difficult due to the dynamic changes occurring during the incubation period. Work is currently ongoing within the OECD Working Party for Manufactured Nanomaterials to develop a test guideline on dissolution testing for metal-containing nanoparticles to support the characterization of dissolution during ecotoxicity testing and the establishment of dose–response relationships [58].

#### Recommendation # 2

Hence, we recommend that the dissolved fraction is characterized over time and for various test concentrations, covering the setup for the aquatic toxicity test conducted. Dissolution should as minimum be determined at the beginning and end of the test, but at shorter intervals for fast dissolving nanoparticles, and for the lowest and highest NP concentration applied. Establishing dissolution rates can support the interpretation of ecotoxicity test results. The measurements should ideally be done in the presence of the test organisms (in situ).

### Nanomaterial surface reactivity is important as toxicity indicator

Some nanomaterials undergo redox reactions, while others are not reactive themselves, but serve as catalysts for chemical reactions, including redox reactions. Regardless of their origin, oxidizing or reducing substances can interfere with the redox balance of cells, by decreasing the level of antioxidants or increasing the production of reactive oxygen species (ROS). This may lead to inflammation and cytotoxicity and has been proposed as a toxic mechanism for nanomaterials [6]. The ability of nanomaterials to generate abiotic ROS, i.e., without the interaction of living cells, has been reported as an nanomaterial-specific characteristic in (eco)toxicological studies [32, 48, 67, 78]. The catalytic activity increases with decreasing nanoparticle size, due to higher specific surface area [33], but also the shape of particles and especially the active step sites have been shown to be of outmost importance for catalytic activity [36].

A study by Sørensen et al. [78] applied a multimethod approach to characterize and link the behavior of platinum nanoparticles (Pt nanoparticles) in test media to the toxic effects induced in two algal species. Pt nanoparticles are widely used as catalysts and, thus, known to possibly be involved in electron transfer with other chemical species. Using the fluorescent dye 2′,7′-dichlorodihydrofluorescein diacetate (H2DCF-DA) as described by Ivask et al. [49], Pt nanoparticles (and PtCl_4_) were found to generate abiotic reactive oxygen species (ROS) in both algal media. This may have contributed to the oxidative stress detected in the two algal species using flow cytometry and the fluorescent probe CellROX green. Pt nanoparticles were also found to generate abiotic ROS when suspended in Milli-Q water, suggesting that ROS were generated on the surface of Pt nanoparticles rather than from interactions with media components. This was further supported by the fact that no abiotic ROS or oxidative stress occurred from the positive control (H_2_O_2_) or the dissolved reference (PtCl4) in one of the media and algal species, whereas Pt nanoparticles caused very clear responses in both these tests. There are many pathways interlinking abiotic/biotic ROS, oxidative stress, DNA damage and cellular toxicity. This challenges the establishment of causality; however, the abiotic ROS generation capacity may serve as toxicity indicator.

#### Recommendation # 3

Abiotic ROS generation capacity should be characterized for nanoparticles possibly undergoing electron transfer interactions with other chemical species, e.g., being redox active or acting as catalysts. Such characterization may assist to explain any toxic effects observed, and/or provide data for future development of nanoparticle SARs.

### A shortened exposure may reduce nanomaterial transformations in ecotoxicity tests and elucidate nanomaterial-specific effects and exposure dynamics

Nanoparticles constitute a great challenge for aquatic toxicity testing due to their highly dynamic behavior in aqueous suspension. As described above, various time-dependent transformation processes including agglomeration/aggregation, sedimentation and especially dissolution cause the exposure concentration to change during the test incubation. This affects the concentration–response relationships and ultimately the validity and reproducibility of aquatic toxicity tests with nanoparticles [38, 39]. Applying a short-term 2 h algal ^14^C-assimilation test, Sørensen & Baun (2015) tested the hypothesis that a shortened exposure period reduces the influence of NP transformation processes on the toxicity outcome and, thus, increases the reproducibility of algal toxicity testing with silver nanoparticles (Ag nanoparticles). The hypothesis was rejected, based on the obtained EC_50_ values for three different Ag nanoparticles, but the approach may be applicable for other types of nanoparticles. However, when suspending the Ag nanoparticles in media 24 h prior to the 2 h testing (aging, see “Using freshly prepared nanomaterial-suspensions may underestimate toxicity” section), a higher degree of reproducibility was obtained. Thus, shortened exposure duration may provide a toxicity snapshot at any given time upon suspension of nanoparticles in media and thereby assist to elucidate the role of the time-dependent NP behavior on toxicity and reproducibility in aquatic toxicity testing of nanoparticles exerting rapid toxic effects and/or dissolution.

The influence of shortened exposure duration was also addressed in a newly developed test with crustaceans (Sørensen et al. 2016). Shortly, daphnids were exposed to a single pulse of 1–3 h duration and then transferred to clean medium where adverse acute and chronic effects were observed during a 48 h and 21 days post-exposure period, respectively. This approach was tested as mean to keep the exposure stable and at the same time disclose acute and chronic effects of Ag nanoparticles and CuO nanoparticles towards *D. magna*. Shortening the exposure duration down to 1–3 h and observing the 48 h post-exposure immobility of daphnids in pure medium offered an acute toxicity test setup that reduced the transformation of nanoparticles during incubation, with comparable sensitivity to disclosing toxicity for Ag nanoparticles and CuO nanoparticles. Pulse exposure is an environmentally relevant exposure scenario for nanoparticles, as they, like many chemical pollutants in general, are discharged into aquatic environments as “pulses” resulting from, e.g., surface runoff after rain events, overflow of wastewater treatment plants, agrochemicals and veterinary pharmaceuticals from agriculture [79]. Also, for Ag nanoparticles and CuO nanoparticles, a pulse exposure test enables more stable exposures and cause acute immobility of *D. magna,* comparable to continuous 24 h exposures [79]. The pulse test thus enables less dynamic exposure conditions and less efforts for monitoring and characterizing the exposure. Also, the chronic effects from 1 to 3 h pulse exposures were monitored over a 21-day post-exposure period. It was found that short-term (1–3 h) exposures to Ag nanoparticles and CuO nanoparticles did not adversely affect reproduction, molting, growth and lethality of *D. magna* to the same extent as in 21 days continuous exposure reproduction tests with the same nanoparticles. However, the pulse test setup allowed for identification of possible nanoparticle-specific effects of CuO nanoparticles, since more pronounced reproductive effects were found for CuO nanoparticles than for CuCl_2_ when the exposure concentration was expressed in terms of the measured dissolved Cu. Retention of CuO nanoparticles in the alimentary canal of the test organisms was suggested as an explanation to this, as the gut content is transferred with the organism to pure medium for the 21 days post-exposure period.

#### Recommendation # 4

A short-term pulse exposure may be applied in addition to the commonly used 48 h exposure, in daphnia toxicity tests with metal and metal oxide nanoparticles prone to dissolution and composed of elements known to be hazardous to the aquatic environment. After the pulse exposure, daphnids are rinsed in medium and moved to clean medium for 48 h, before the immobility or lethality is registered. The added benefit of this approach is minimized efforts needed to monitor and characterize nanoparticles during the exposure period. The application potential for other less toxic nanoparticles remains to be studied.

### Using freshly prepared nanomaterial-suspensions may underestimate toxicity

In the already mentioned study by Sørensen and Baun (2015), another hypothesis was tested: that the guideline approach of using freshly prepared test solutions for aquatic toxicity testing may not be appropriate for nanoparticle suspensions. This is because the transfer of nanoparticles from stock suspensions into medium may be the starting point for a series of time-dependent transformation processes of nanoparticles. It was found that short-term (2 h) algal toxicity testing in which Ag nanoparticles were added the ISO algal test medium 24 h prior to toxicity testing (24 h aging) resulted in a higher degree of reproducibility and clearer concentration–response relationships than when freshly prepared suspensions were used. Furthermore, the length of the aging step influenced the toxicity test outcome, as toxicity increased to a maximum after 48 h aging, and then declined with further aging until 7 days. This toxicity pattern could be explained by the dissolution and agglomeration/aggregation kinetics of Ag nanoparticles in the algal medium (Sørensen and Baun 2015).

#### Recommendation # 5

For nanoparticles that dissolve in testing media, acute toxicity tests should be conducted not only using a freshly prepared suspension of nanoparticles in test medium, but also an aged suspension where nanoparticles are added to the media for example 1–3 days prior to testing. The relevant aging duration may differ for various media and nanoparticles, and should ideally be determined on a case-by-case basis. However, the duration of the aging step should be kept within a feasible and reproducible timeframe and, thus, we suggest maximum one week. This aging step may increase or decrease toxicity. Regardless of the outcome of the aging step, an indication of nanoparticle transformation processed and their influence on toxicity will be obtained.

### It is important to distinguish between physical and chemical effects in aquatic toxicity tests

The standard aquatic toxicity tests utilized for dose–response assessment of nanoparticles were originally developed to reflect direct toxic effects of soluble chemicals. Nanoparticles are inherently different from soluble chemicals, as they are suspended in media rather than dissolved, and the effects arising from contact between the nanoparticles and the organism may result from physical interferences, rather than direct toxicity [74]. The adhesion of nanoparticles to test organisms has been frequently reported [e.g., 1, 2, 10, 13, 16, 38, 39, 55, 66; Sørensen et al. 2015; 81]. In immobilization tests with crustaceans, the nanoparticle adhesion to antennas and exoskeleton of the organism may mechanically impair their mobility, as observed for platinum nanoparticles (Sørensen et al., 2015) and TiO_2_ [16]. Sørensen et al. (2015) concluded mortality to be a more appropriate endpoint than immobility, in order to account for toxicity rather than physical restriction. However, it should be acknowledged that immobility is a more sensitive endpoint and that mortality may in fact be caused by nanoparticle adhesion. No matter whether immobility or mortality is used as endpoint, it is strongly recommended to supplement the observation with visual inspections of immobile and dead animals to take the possibility of physical effects into account. In algal growth inhibition tests, nanoparticle adhesion to alga may inhibit growth by influencing the nutrient or light availability (shading). Several studies have investigated the influence of nanoparticle shading using setups that separate the algae from the nanoparticle suspensions. Such studies have both confirmed and rejected the influence of shading on algal growth inhibition [2, 38, 46, 71, 82]. Significant shading has been identified for Pt nanoparticles of relatively low toxicity with EC_50_ values in the higher end of the CLP classification range for aquatic toxicity (10–100 mg/L) [78]. This exemplifies the need to account for shading effects, even at relatively high concentrations, when conducting aquatic toxicity tests for dose–response assessment purposes. Under the current standard testing scheme, physical effects are considered a confounding factor rather than actual toxic effects. Thus, physical effects need to be accounted for and eliminated as best possible. It could be argued that some physical effects of nanoparticles constitute environmentally relevant effect mechanisms different from what is known for dissolved chemicals. For example, it may also be argued that certain physical effects (e.g., algal shading caused by turbid suspensions) are artefacts, whereas others (such as algal shading on a cellular level caused by adhesion of nanoparticles to the cell surface) should be considered NP-specific effects and hence not a confounding factor. As a minimum, however, it is important to account for the different types of physical and chemical effects that contribute to the overall measured effect in the test.

#### Recommendation # 6

The impact of shading must be accounted for when conducting algal growth rate inhibition tests with nanoparticles as indicated by ECHA in their updated version of the nanospecific appendix to R.7b. In certain cases, nanoparticles may be expected to reduce light availability to the alga, i.e., for nanoparticles that (1) produce dark/turbid media suspensions, (2) adhere to algal surfaces, or (3) have relatively low toxicity and, therefore, require to be tested in exposure concentrations in the upper end of the CLP classification range (10–100 mg/L). Shading effects are most easily studied in testing setups where the NP suspensions are physically contained separately and placed in between the light source and the container with algae. While such setups fail to disclose localized shading caused by nanoparticles adhering to algal cells, they are practically feasible with only minor additional testing required. For daphnia testing, nanoparticles that undergo agglomeration/aggregation and sedimentation or visibly adhere to the exterior of the daphnia are likely to induce physical effects causing immobility and/or death. This behavior often occurs when nanoparticles in powder-form are suspended in test media. Also, a mesh can be inserted in test beakers to keep daphnids from contact with nanoparticles deposited on the bottom of the test beakers.

### Toxicity and uptake are feeding dependent

In a study of the acute and chronic toxicity of 30 nm citric acid stabilized Ag nanoparticles to *Daphnia magna*, Mackevica et al. [54] studied how food availability influenced toxicity. The tests by Mackevica et al. [54] were done according to the OECD *Daphnia magna* 21-day reproduction tests [57] and the experiments were carried out as static renewal tests with exposure concentrations from 10 to 50 μg Ag/L. Test animals were fed with green algae *Pseudokirchneriella subcapitata* in a low food regime (as suggested by OECD guideline 211) and a high food regime (three times the amount applied for low food conditions). For acute toxicity, the concentration–response curves and the 48 h EC50 values obtained in the acute toxicity tests with Ag nanoparticles (25–300 µg Ag/L) clearly showed a clear decrease in toxicity with more food provided (low food regime compared to high food regime). The survival, growth and reproduction of *D. magna* were affected with similar concentration–response patterns for both food treatments during the 21-day incubation in the chronic toxicity tests. In the case of low food treatment, an increased mortality was observed for concentrations higher than 20 μg/L, whereas for high food treatment similar effects occurred at concentrations above 40 μg/L. Mackevica et al. [54] observed that although daphnids exposed to Ag nanoparticles and usual food treatment survived when exposed at the highest Ag nanoparticles concentration, they did not produce any offspring while daphnids exposed to the same Ag nanoparticles exposure concentration and the high food treatment were still able to reproduce even at 50 µg/L. It should be noted that the addition of food could influence the agglomeration/aggregation pattern and the potential for increased uptake due to adhering nanoparticles to, e.g., algae as a food source for *D. magna*. Indeed, Sakka et al. [70] showed that the addition of food interfered with the stability of Ag nanoparticles such that sterically stabilized Ag nanoparticles were more stable compared to charge stabilized Ag nanoparticles in the presence of algae [70]. In terms of potential differences in uptake due to addition of food, Skjolding et al. [72] found that feeding resulted in a net decrease in uptake of AuNP but also gave rise to a higher deviation in uptake which could imply sorption of AuNP onto algae. The presence of algae resulted in a very fast excretion of ingested particles during the depuration period in clean medium without AuNP added.

#### Recommendation # 7

Due to the fact that toxicity has been observed to be feeding dependent and related to uptake of nanoparticles, an accurate accounting for food abundance in all tests is required. It is recommended that tests are carried out at with at least two different food levels. This could be with food availability according to OECD TGs (for instance OECD guideline 211) and with food abundance three to five times higher than this. Furthermore, uptake studies lasting for 24 h (as described in, e.g., Skjolding et al. [72] and Sakka et al. [70]) could reveal important toxicity mechanism by relating uptake and toxicity in long-term reproduction studies of nanoparticles.

### Uptake and depuration depend on nanomaterial functionalization

Quantification of organism uptake and depuration is key for assessing bioaccumulation and for nanoparticles; it seems that the nanoparticle functionalization governs these processes. The study by Skjolding et al. [73] investigated the influence of surface functionalization on uptake and depuration behavior of ZnO nanoparticles, ZnO-OH nanoparticles and ZnO-octyl nanoparticles in of *D. magna*. Whereas no uptake was observed for Zn-OH nanoparticles during 24 h incubation, a rapid initial uptake was observed for ZnO nanoparticles and ZnO-octyl nanoparticles. It was, furthermore, found that the body burdens were higher for functionalized ZnO-octyl nanoparticles compared to non-functionalized ZnO nanoparticles showing that functionalization of the nanoparticles highly influenced the uptake and depuration in the animals. The uptake of non-functionalized ZnO nanoparticles was observed to be 4.6 times and 2.3 times higher compared to ZnCl_2_ and ZnO bulk (<5 µm), respectively. Even though *D. magna* body burdens were 9.6 times and 47 higher for ZnO nanoparticles and ZnO-octyl nanoparticles, respectively, than toxic levels reported for zinc, Skjolding et al. [73] did not observe increased mortality after the exposure to the Zn-containing nanoparticles. In summary, the results by Skjolding et al. [73] show that differently functionalized nanoparticles exhibit different bioavailability to *D. magna* even though the core material is the same. Similar findings were found for differently functionalized Au nanoparticles (citrate and MUDA), showing different uptake and depuration behavior in *D. magna* after 24 h of uptake [72]. Furthermore, after depuration for 24 h without addition of food a marked difference in residual body burden of Au was observed for the different functionalizations. While a plethora of different functionalizations exists, systematic studies of the influence of these on uptake and depuration in aquatic organisms are lacking. Furthermore, with different functionalizations follows different agglomeration behavior, thus introducing other variables that could influence the uptake, e.g., size [72, 73]. In a similar study by Wray and Klaine [83], an addition of amine groups to Au nanoparticles by granting them a negative surface charge resulted in higher ingestion efficiencies in *D. magna*. Thus, differences in surface charge resulting from the functionalization could naturally influence the ingestion rate hence increasing the number of nanoparticles in the gut. Feswick et al. [35] also found that negatively charged quantum dot nanoparticles were taken up more than positively charged nanoparticles in *D. magna*, which could be correlated with the increased ingestion efficiency due to the negative charge of the nanoparticles. Similarly, Sakka et al. [70] found higher uptake of charge stabilized Ag nanoparticles (citrate) compared to sterically stabilized Ag nanoparticles (NM-300 K) in *D. magna* after 24 h uptake at nominal concentration of 10 µg Ag/L. Consequently, the potential for functionalization to alter behavior in terms of agglomeration but also in terms of surface charge has to be considered in relation to both the intrinsic properties of the nanoparticles but also in the context of the test organisms feeding traits.

#### Recommendation # 8

Uptake and depuration of nanoparticles in test organisms have to be determined for each commercialized functionalization of the nanoparticles as differently functionalized nanoparticles can exhibit different bioavailability to, e.g., *D. magna* even though the core material is the same. If systematic studies are carried on the influence of nanoparticle functionalizations on uptake and depurations, these processes may eventually pave the way for future development of nanospecific QSARs.

### Trophic transfer is an important uptake pathway for nanomaterials

In the study by Skjolding et al. [73], the trophic transfer of ZnO NP and Zn-octyl NP from daphnids (*D magna*) to zebrafish (*Danio rerio*) was studied. It was found that uptake of both ZnO NP and Zn-octyl NP reached values more than ten times higher than the levels obtained through aqueous exposure in other studies. For dietary exposure (daphnia pre-exposed to 1 mg Zn/L for 24 h) of ZnO NP and ZnO-octyl NP, body burdens as high as 880 ± 180 and 2170 ± 410 mg Zn/kg dry weight were found, respectively. In comparison, Yu et al. [84] found that uptake of ZnO NP and bulk ZnO through aqueous exposure to 10 mg Zn/L for 96 h resulted in levels around 40 and 45 mg Zn/kg dry weight, respectively. Consequently, the body burdens in zebrafish reported by Skjolding et al. [73] through dietary exposure were approximately 20- and 50-fold higher for ZnO nanoparticles and bulk ZnO compared to water exposure. Similarly, higher body burdens of Ag were observed after exposure to Ag nanoparticles through contaminated food than for aqueous exposures [3]. Contrary, Ates et al. [4] found lower body burdens for dietary exposure of CuO nanoparticles and ZnO nanoparticles compared to aqueous exposure. However, a marked difference in dissolution was observed between the two exposure routes. CuO nanoparticles and ZnO nanoparticles showed 98 and 55% dissolution (in zebrafish media), respectively, in the aqueous exposure compared to <5% in the dietary exposure for both nanoparticles. To quantify differences in localization and minimizing the confounding factor of dissolution, Skjolding et al. [76] exposed juvenile zebrafish (*D. rerio*) to Au nanoparticles. After two days incubation, a significantly higher total body burden was observed for the dietary exposure compared to the aqueous exposure. Using Light Sheet Microscopy (LSM), it was observed that the dietary exposure resulted in a strong fluorescent signal from the stomach and intestines, while the aqueous exposure was less pronounced in the stomach and intestines. Due to overlap of the excitation wavelengths with the background fluorescence of the zebrafish, the spatial resolution was not sufficient to observe differences in other compartments of the zebrafish [76]. The trends observed correspond well with differences between aqueous and dietary exposure reported in the literature [3, 4, 17, 52]. It is important to highlight that trophic transfer as explained throughout this paragraph is mainly of importance for slow or non-dissolving nanomaterials.

#### Recommendation # 9

Trophic transfer is a topic of high importance to the dose–response assessments of nanoparticles and has to be taken into account as both exterior bound and/or accumulated nanoparticles in prey organisms will be available for predator organisms. It is important to note that trophic transfer mainly be of high importance for slow or non-dissolving nanomaterials. Furthermore, differences in internal localization patterns have been observed for aqueous and dietary exposure for different organisms.

### Data selection for dose–response assessment derivation should be structured, reproducible and transparent and support use on non-guideline data

In the technical guidance document for safety assessment of chemicals in support of REACH, the purpose of the dose–response evaluation during hazard assessment is to evaluate available ecotoxicity data “for use in classification and labeling and derivation of the PNECwater (Predicted No Effect Concentration for water) and for determination of the toxicity (T) criterion in the PBT assessment” [27]. The data used for dose–response assessment must undergo a critical evaluation for their regulatory relevance and reliability. It is current practice that ecotoxicological data are considered more valid for regulatory use if obtained according to accepted and validated test guidelines, preferably also following good laboratory practice (GLP). However, engineered nanoparticles are known to behave very differently in ecotoxicity test systems compared to soluble chemicals, for which most guidelines were intended. For this reason, non-standard tests, or tests following modified test guidelines, can provide valuable information and should not per se be considered less reliable for dose–response assessment purposes. To assist the expert judgement needed to assess the adequacy of both guideline and non-guideline ecotoxicity data for nanoparticles for regulatory use, Hartmann et al. [42] have developed a structured, transparent and reproducible science-based approach. The approach is based on 21 data reliability evaluation criteria, taking into account the testing challenges and characterization requirements that are associated with nanomaterial ecotoxicity testing. The criteria can be used to make transparent evaluations of data reliability for all types of nanoparticles and aquatic ecotoxicity studies. The approach can be used to make a qualitative or quantitative data evaluation depending on the specific scoring system that is applied by the evaluator. The result of the evaluation is a classification of the specific study as nRi1 (reliable without restrictions), nRi2 (reliable with restrictions), nRi3 (not reliable) or nRi4 (not assignable). In combination with the so-called CRED criteria for evaluation of data relevance (published by Moermond et al. 56]), an overall evaluation of data adequacy for nanomaterial ecotoxicity studies can be made.

#### Recommendation # 10

It is recommended that new nanospecific guidance is developed as an appendix to the supporting reference guidance in Chapter R.4 on Evaluation of available information. This guidance should provide a transparent and science-based method to assess the reliability and relevance of data on NP (eco) toxicity data. For ecotoxicity, data reference can be made to the methods developed by Hartmann et al. [42] and Moermond et al. [56].

## Concluding remarks

The European Chemical Agency (ECHA) is in the process of revising its guidance documents on how industry is to complete chemical safety assessments to address the challenges that nanoparticles pose for ecotoxicological testing. Based on an analysis of the EnvNano findings in the light of the guidance updates planned by ECHA, we conclude that ECHA has made a lot of progress in regard to updating its guidance documents on sample preparation and characterization. However, there is still room for more specific guidance on how the nanospecific sample preparation and characterization techniques and methods are to be applied as well as in regard to how to perform and interprete the results of ecotoxicological testing of nanoparticles.

Some key EnvNano findings, such as the need to characterize dispersion and dissolution rates in stock and test media, have been addressed in the updated guidance. However, it has to be further specified that multiple characterization methods should be applied to describe state of dispersion and dissolution over time and for various test concentration. More detailed information is called for on the specific characterization methods and techniques available and their pros and cons. The importance of considering the impact of shading is also mention in the ECHA guidance, but limited guidance is provided on how to account for this when conducting algal growth rate inhibition tests with nanoparticles. In general, more specific guidance is needed on methods to discriminate between different types of effects including physical effects, effects caused by dissolved ions and nanoparticle effects. In algal tests, shading can be studied in testing setups where the nanoparticle suspensions are physically contained separately and placed in between the light source and the container with algae. Although such tests cannot disclose localized shading caused by nanoparticles adhering to algal cells, they are practically feasible with only minor additional testing required. In daphnia tests, a mesh can be inserted in test beakers to keep daphnids from contact with nanoparticles deposited on the bottom of the test beakers, thereby facilitating the discrimination between different types of effects.

Based on the project findings, we recommend that existing algal tests are *supplemented* with tests where nanoparticle suspensions are aged for 1-3 days for nanoparticles that dissolve in testing media. Likewise, for daphnia tests we suggest to *supplement* with tests where (a) exposure is shortened to a 3 h pulse exposure followed by 48 h post-exposure period in clean medium for tests with environmentally hazardous metal and metal oxide nanoparticles prone to dissolution; and (b) food abundance is three to five times higher than normal, respectively. Determination of uptake and depuration of nanoparticles in test organisms, furthermore, has to be taken into account for each commercialized functionalization of the nanoparticles and trophic transfer as both exterior bound and/or accumulated nanoparticles in prey organisms will be available for predator organisms, thus potentially increasing the toxicity compared to aqueous exposure.

Finally, we recommend that nanoparticle ecotoxicity data are evaluated with regard to regulatory adequacy using a systematic and transparent approach, where expert judgement is assisted by a science-based framework for assessing study reliability, as developed by Hartmann et al [42]. For this, we have identified a need for a new nanospecific appendix to Chapter R.4. on evaluation of available information.
